# The effect of DASH diet on components of metabolic syndrome: a systematic review and meta-analysis of randomized controlled trials

**DOI:** 10.3389/fnut.2026.1738410

**Published:** 2026-05-11

**Authors:** Pengyu Zhao, Wenyun Fu, Lanxin Cui, Ling Lin

**Affiliations:** 1 The First School of Clinical Medicine, Hainan Medical University, Haikou, Hainan, China; 2 Department of Cardiology, Sanya Central Hospital (Hainan Third People’s Hospital), Hainan Medical University, Sanya, Hainan, China

**Keywords:** DASH diet, dietary approaches to stop hypertension, meta-analysis, metabolic syndrome, randomized controlled trials

## Abstract

**Background:**

Metabolic syndrome (MetS) has emerged as an increasingly significant global health issue. The Dietary Approaches to Stop Hypertension (DASH) diet is recommended for managing MetS. However, existing meta-analyses often focus on individual MetS components, without providing a comprehensive evaluation of the DASH diet’s effects on the full spectrum of MetS components. Therefore, we conducted a systematic review and meta-analysis of randomized controlled trials evaluating the effects of the DASH diet on various components of MetS.

**Methods:**

We performed a comprehensive search of PubMed, Web of Science, Embase, and Cochrane Library databases, covering studies published until April 6, 2025. We included RCTs that evaluated the effects of the DASH diet on MetS components such as waist circumference (WC), blood pressure (BP), fasting blood glucose (FBG), high-density lipoprotein cholesterol (HDL-C), triglycerides (TG), and Homeostasis Model Assessment-Insulin Resistance (HOMA-IR). Sensitivity analyses were conducted to assess result stability, and funnel plots were used to assess publication bias, with Egger’s test applied for significance (*p* < 0.05). Additionally, we used the GRADE approach to assess the certainty of evidence.

**Results:**

A total of 14 studies involving 1,062 participants were included. The meta-analysis showed that, compared with non-DASH diets, the DASH diet significantly reduced WC (MD = −2.33 cm, 95% CI: −3.10 to −1.57), systolic BP (MD = −5.52 mmHg, 95% CI: −6.83 to −4.21), diastolic BP (MD = −3.93 mmHg, 95% CI: −4.76 to −3.10), TG (MD = −16.57 mg/dL, 95% CI: −23.53 to −9.61), HDL-C (MD = 1.44 mg/dL, 95% CI: 0.20 to 2.68), and HOMA-IR levels (MD = −0.71, 95% CI: −1.10 to −0.31). However, no significant difference was observed in FBG (MD = −0.91 mg/dL, 95% CI: −4.51 to 2.68). The GRADE approach for assessing certainty of evidence indicated that, with the exception of FBG and HDL-C where the certainty of evidence was low, the overall certainty of evidence for the remaining outcome measures was rated as moderate quality.

**Conclusion:**

The DASH diet appears to have a positive effect on components of MetS and can be considered an effective dietary intervention for MetS patients.

**Systematic review registration:**

CRD420251060235.

## 1 Introduction

Metabolic syndrome (MetS), also known as Syndrome X, is a major risk factor for cardiovascular disease worldwide (1). Characterized primarily by insulin resistance, MetS often coexists with central obesity, hypertension, dyslipidemia, impaired glucose metabolism, and chronic inflammation (2). Diagnostic criteria for MetS are based on guidelines from major organizations, including the World Health Organization (WHO), the National Cholesterol Education Program Adult Treatment Panel III (NCEP-ATP III), and the International Diabetes Federation (IDF) (3). Of these, the NCEP-ATP III and IDF criteria are most widely used, both emphasizing central obesity, with waist circumference as a key measure. In contrast, the WHO criteria are more stringent, focusing primarily on insulin resistance (4).

MetS components typically include abdominal obesity, hypertension, dysregulated glucose metabolism, and dyslipidemia (elevated TG levels and reduced HDL-C levels) (5). Global prevalence varies with the diagnostic criteria applied, ranging from 12.5 to 31.4% (6). However, regardless of the specific criteria, MetS remains highly prevalent and continues to rise. MetS significantly increases the risk of severe conditions, including cardiovascular disease and type 2 diabetes (1, 7). Studies show that MetS approximately triples the risk of cardiovascular disease and increases the likelihood of developing type 2 diabetes by fivefold (8). Consequently, MetS is a major contributor to all-cause mortality, with individuals diagnosed with MetS facing a 46% higher risk of death compared to those without it (9). As a preventable condition, MetS is closely associated with unbalanced dietary habits (10). Research confirms that preventive measures such as adopting a healthy diet can effectively prevent MetS (11). Lifestyle modifications, particularly dietary changes, are the primary therapeutic strategy for treating and managing MetS, as recommended by the guidelines (12). Therefore, establishing healthy eating habits is a key intervention to effectively reduce the incidence of MetS (13).

The Dietary Approaches to Stop Hypertension (DASH) diet, introduced in 1997, has proven effective in lowering BP and improving lipid profiles by increasing potassium, magnesium, calcium, and dietary fiber, while reducing sodium, saturated fat, and refined sugar intake (14, 15). In addition, the high intake of fruits and vegetables in the DASH diet provides a wealth of antioxidants, which are also crucial for correcting glucose and insulin abnormalities. The DASH diet may be associated with MetS (16). Originally developed to combat hypertension, evidence now suggests that the DASH diet also benefits individuals with MetS by improving BP, lipid profiles, glucose metabolism, and other cardiovascular risk factors (12, 17). Recent studies have further investigated its role in managing MetS components. For instance, Liu et al. found that higher adherence to the DASH diet is inversely associated with the risk of MetS, particularly affecting WC, FBG, SBP, DBP, TG, and HDL-C (13).

However, the current body of randomized controlled trials (RCTs) evaluating the DASH diet for MetS is largely comprised of small-sample studies, and the overall strength of evidence remains limited. Moreover, existing meta-analyses have typically focused on only selected components of MetS, such as BP and HDL-C (18), leaving a notable evidence gap regarding a systematic and comprehensive assessment of the DASH diet across the full spectrum of core components, namely WC, BP, FBG, HDL-C, HOMA-IR, and TG. Accordingly, we conducted a systematic review and meta-analysis to assess the effects of the DASH diet on key features of MetS. By synthesizing high-quality evidence, this study will strengthen the evidence base for personalized dietary management in patients with MetS.

## 2 Methods

### 2.1 Protocol and registration

This systematic review and meta-analysis was prospectively registered in PROSPERO (Registration number: CRD420251060235) (19).

### 2.2 Search strategy

We performed a systematic search in PubMed, Web of Science, Embase, and the Cochrane Library for studies published up to April 6, 2025. The search included terms like “DASH Diet,” “Metabolic Syndrome,” and “RCTs.” We also screened the reference lists of relevant articles to identify additional studies. Only English-language studies were included. The detailed search strategy was provided in Supplementary Table S1.

### 2.3 Eligibility criteria

Inclusion criteria were: (a) randomized controlled trials (RCTs), (b) adult participants (≥18 years) diagnosed with MetS, (c) intervention with any form of DASH diet, (d) control groups receiving usual diets, conventional diets, routine dietary advice, or other non-specific interventions rather than another structured dietary pattern, and (e) primary outcomes including waist circumference (WC), blood pressure (BP), fasting blood glucose (FBG), HDL-C, HOMA-IR, or triglycerides (TG). Exclusion criteria included non-randomized trials, animal studies, case reports, reviews, conference abstracts, and studies with unavailable or non-convertible data. Studies directly comparing the DASH diet with other specific dietary patterns (e.g., the Mediterranean diet) were also excluded.

### 2.4 Data extraction

Two authors (PY Zhao and WY Fu) independently extracted data, which was verified by a third reviewer (LX Cui). Extracted data included study characteristics (e.g., first author, year, sample size, age), treatment methods, study design, and clinical outcomes. For studies reporting different units, we standardized values to consistent units (e.g., mmol/L to mg/dL for glucose, TG, and HDL-C). We contacted corresponding authors for missing data and resolved discrepancies through discussion, consulting a third reviewer when necessary.

### 2.5 Risk of bias

We assessed the risk of bias in included RCTs using the Cochrane Risk of Bias Assessment Tool (20), covering domains such as detection bias, selection bias, attrition bias, and reporting bias. Each study was classified as having “high risk,” “low risk,” or “uncertain” bias in each domain (21). Blinding was often not feasible due to the nature of dietary interventions, and thus, participant blinding was not prioritized in the quality assessment.

### 2.6 Data synthesis

Continuous variables were standardized using standard deviation (SD), and net change was calculated with the formula:

$$\mathrm{SD}=\sqrt{\left(\begin{array}{l} \mathrm{SD}(\text{baseline})2+\mathrm{SD}(\text{endpoint})2-2R\\ \times \mathrm{SD}(\text{baseline})\times \mathrm{SD}(\text{endpoint}) \end{array}\right)}$$

If necessary, the correlation coefficient (R) was assumed to be 0.5 when data were unavailable. We calculated mean differences (MD) and 95% confidence intervals (CI) for continuous outcomes. Heterogeneity was assessed with the I^2^ statistic, and if I^2^ > 50%, a random-effects model was applied.

Subgroup analyses were conducted according to intervention duration and participants’ age. Specifically, intervention duration was stratified into ≥12 weeks and <12 weeks, while age was categorized as ≥50 years and <50 years. These stratifications were prespecified to further elucidate the potential influence of varying intervention exposure periods and age-related factors on the primary outcome of WC.

Sensitivity analyses were conducted to assess the stability of the results, and funnel plots were used to assess publication bias, with Egger’s test applied for significance (*p* < 0.05). Meta-analysis was conducted using RevMan 5.4 and STATA 16.0.

### 2.7 Certainty of evidence

We evaluated the certainty of evidence using the GRADE approach, considering factors such as risk of bias, inconsistency, indirectness, imprecision, and publication bias. The overall certainty was classified as very low, low, moderate, or high (22).

## 3 Results

### 3.1 Study selection

**Figure 1:**
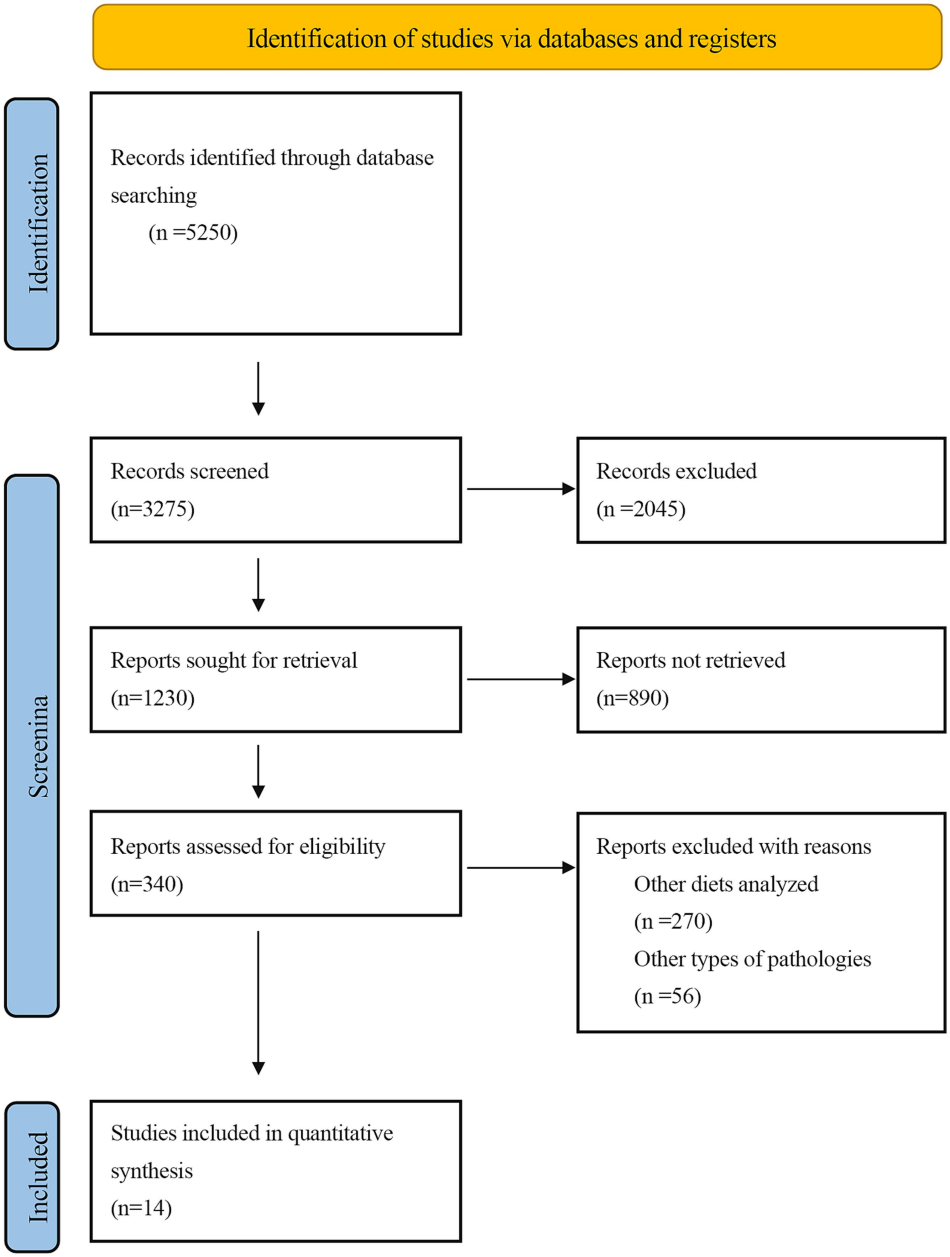
Flow diagram of the eligibility process of included studies.

The electronic search yielded 5,250 articles, of which 14 met the eligibility criteria for full-text review, as shown in the flow diagram (Figure 1). Ultimately, 14 RCTs were included in this systematic review and meta-analysis (23–36).

### 3.2 Study characteristics

Table 1 summarizes the 14 studies included in this meta-analysis, conducted across several countries, including the United States and Iran. Individual study sample sizes ranged from 44 to 126 participants, with ages between 22 and 62 years, for a total of 1,062 participants. All participants had MetS, and common coexisting conditions included hypertension, type 2 diabetes, and overweight or obesity. In every study, the intervention group followed the DASH diet, while control groups continued their usual diet or received other dietary approaches. Intervention duration ranged from 6 weeks to 6 months.

**Table 1:** Characteristics of the included studies.

Author, year	Country	Sample size (T/C)	Age (y)	Intervention diet	Control diet	Duration	Outcomes
Asemi et al. (32)	Iran	24/24	T: 22.1 ± 3.2; C: 24.7 ± 6.0	Adopt the DASH diet to increase dietary fiber and reduce fat and salt intake.	Participants maintained their regular diet; no specific restrictions apply.	8 weeks	TG, HDL-C
Azadbakht et al. (35)	Iran	38/40	T: 41.5 ± 12.5; C: 41.3 ± 12.9	The DASH diet is characterized by high intake of fruits, vegetables, whole grains, and low-fat dairy products; reduced consumption of saturated fats and cholesterol; increased intake of minerals such as potassium, calcium, and magnesium; and an emphasis on dietary fiber.	Participants did not receive dietary intervention and were only instructed to maintain their usual dietary patterns. The nutrients consumed were similar to the typical dietary patterns observed in the adult population of Tehran, Iran.	6 months	BP, FBG, WC, TG, HDL-C
Azadbakht et al. (29)	Iran	31/31	No info	Follow the DASH diet, rich in fruits, vegetables, whole grains, and low-fat dairy products, while low in saturated fat, total fat, cholesterol, refined grains, and sweets, with a sodium intake of 2,400 milligrams per day.	This diet is a standard diet with a sodium intake of 2,996 milligrams per day, and does not emphasize adjustments to specific food types or nutrients.	8 weeks	BP, FBG, WC, TG, HDL-C
Blumenthal et al. (33)	USA	46/49	T: 51.8 ± 10.0; C: 51.8 ± 9.0	Participants followed the DASH eating pattern, reducing total fat (27%), saturated fat (6%), and cholesterol intake while increasing dietary fiber, potassium, magnesium, and calcium consumption.	Participants followed a typical American diet, with fat accounting for 34% of total calories and protein for 15%, while potassium, magnesium, calcium, and fiber intakes were close to the average levels for the U. S. population.	4 months	BP
Blumenthal et al. (34)	USA	46/49	T: 51.8 ± 10.0; C: 51.8 ± 9.0	Follow the DASH diet plan, which emphasizes a high-fiber diet rich in fruits, vegetables, and low-fat dairy products, while being low in fat and sodium.	Participants did not receive any dietary intervention and continued to maintain their usual lifestyle and eating habits.	4 months	TG, FBG, HDL-C
Epstein et al. (36)	USA	48/48	No info	Participants followed the DASH eating pattern, increasing their intake of fruits, vegetables, and low-fat dairy products while reducing their consumption of fat, saturated fat, sweets, and sodium.	Participants maintained a typical American dietary pattern, characterized by high intake of fat, cholesterol, and sodium, and low consumption of fruits, vegetables, and low-fat dairy products.	4 months	BP
Hashemi et al. (25)	Iran	35/40	T: <50 years; C: <50 years	Daily intake of 1,700 calories, including at least three servings of whole grains, three servings of vegetables, two servings of fruit, low-fat dairy products, moderate amounts of nuts, seeds, and legumes, while limiting meat and fat consumption.	Daily intake of 1,700 calories, comprising standard dietary components, with no specific restrictions on sodium, fat, or sugar intake.	12 weeks	BP
Hikmat et al. (31)	USA	35/32	T: 44.5 ± 10.5; C: 45.4 ± 11.4	The DASH diet is rich in fruits, vegetables, and fiber, incorporates low-fat dairy products, and reduces intake of saturated fats and total fats.	The diet is typical of American eating patterns, with intakes of carbohydrates, fat, protein, and fiber close to the median for U. S. consumers. Potassium, magnesium, and calcium intakes are around the 25th percentile.	8 weeks	BP
Kucharska et al. (26)	Poland	64/62	T: 61.3 ± 7.9; C: 58.1 ± 8.5	Follow a personalized DASH diet.	Standard dietary intervention only.	3 months	WC, FBG, HOMA-IR
Paula et al. (28)	Brazil	20/20	T: 61.8 ± 8.1; C: 62.5 ± 8.8	Adopting the DASH diet intervention improves blood pressure by reducing sodium intake.	Did not receive the DASH dietary intervention; maintained their usual diet and activity level.	4 weeks	BP, FBG, WC, TG, HDL-C
Rashidbeygi et al. (30)	USA	50/48	T: 40.3 ± 9.7; C: 40.6 ± 11.1	Adopt a calorie-restricted DASH diet that emphasizes whole foods, high fiber, low sodium intake, and appropriate portion control to promote weight management and improve cardiovascular and metabolic health.	Adopting a low-calorie diet focuses on calorie restriction but does not specifically emphasize food variety, nutrient density, or portion control.	16 weeks	FBG, HOMA-IR
Razavi Zade et al. (27)	Iran	30/30	T: 39.7 ± 7.3; C: 42.8 ± 10.6	Follow the DASH diet plan, which is low in sugar and high in fiber, rich in antioxidants, calcium, magnesium, vitamin C, and polyunsaturated fatty acids. It aims to improve metabolic profiles, inflammatory markers, and oxidative stress.	Maintained a regular diet without specific nutritional intervention.	8 weeks	WC, TG, HDL-C, FBG, HOMA-IR
Sangouni et al. (23)	Iran	30/30	T: 44.4 ± 6.1; C: 45.6 ± 7.1	The DASH diet emphasizes high fiber intake and is rich in fruits, vegetables, whole grains, and low-fat dairy products, while limiting saturated fats, cholesterol, refined grains, and sugary beverages. Its macronutrient distribution is as follows: 50–55% carbohydrates, 15–20% protein, and 30% fat.	Follow a healthy diet that includes a balanced variety of foods, with the same macronutrient distribution as the intervention group, but lower in fiber and micronutrients compared to the DASH diet.	12 weeks	BP, WC, TG, HDL-C
Sorić et al. (24)	Croatia	33/34	T: 53.2 ± 8.9; C: 50.7 ± 8.0	Follow the DASH eating plan, which emphasizes low-fat dairy products, whole grains, lean meats, fruits, and vegetables, while reducing sodium intake.	Maintained a regular diet without specific nutritional intervention; dietary composition differed from that of the intervention group.	3 months	BP, WC, TG, HDL-C, FBG

T, treatment group; C, control group; WC, waist circumference; SBP, systolic blood pressure; DBP, diastolic blood pressure; TG, triglycerides; HDL-C, high-density lipoprotein cholesterol; FBG, fasting blood glucose; HOMA-IR, Homeostasis Model Assessment–Insulin Resistance; DASH, Dietary Approaches to Stop Hypertension.

### 3.3 Quality assessment

**Figure 2:**
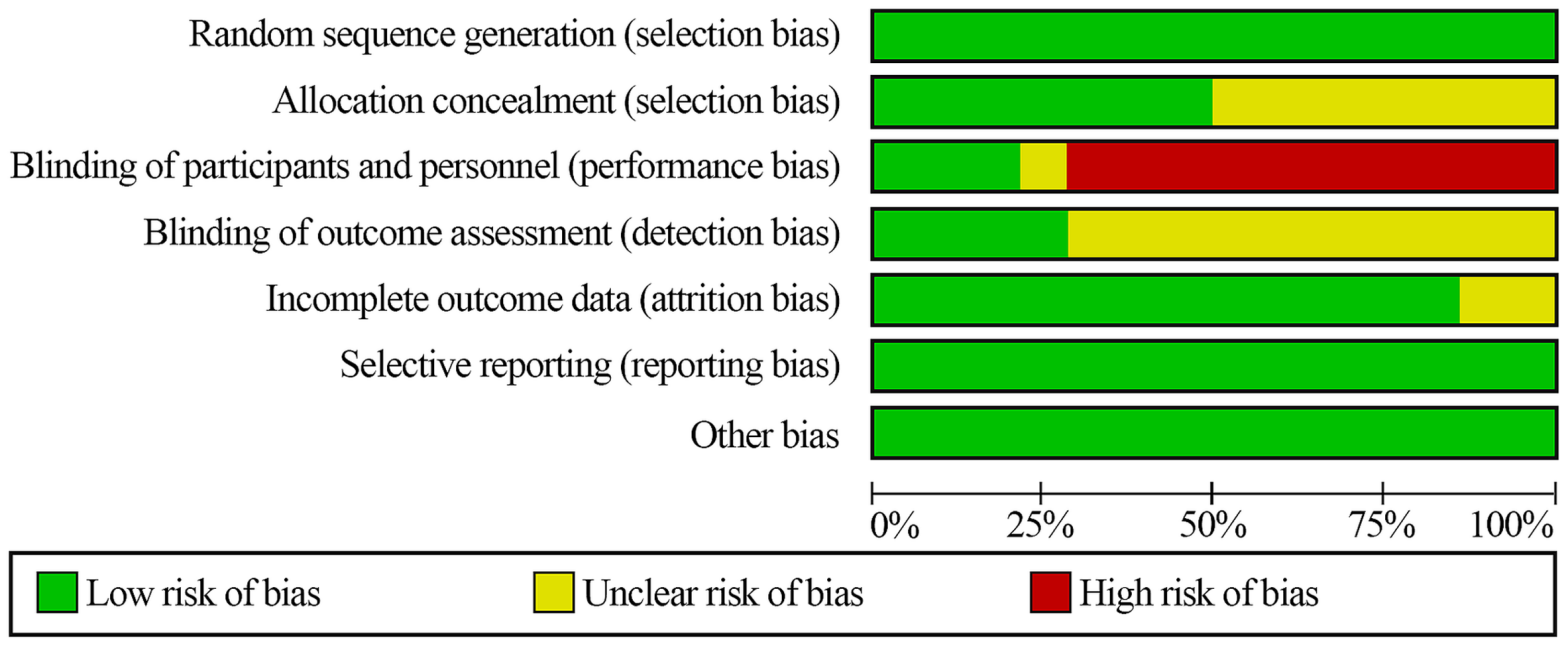
Risk of bias summary review authors’ judgments about each risk of bias item for each included study.

**Figure 3:**
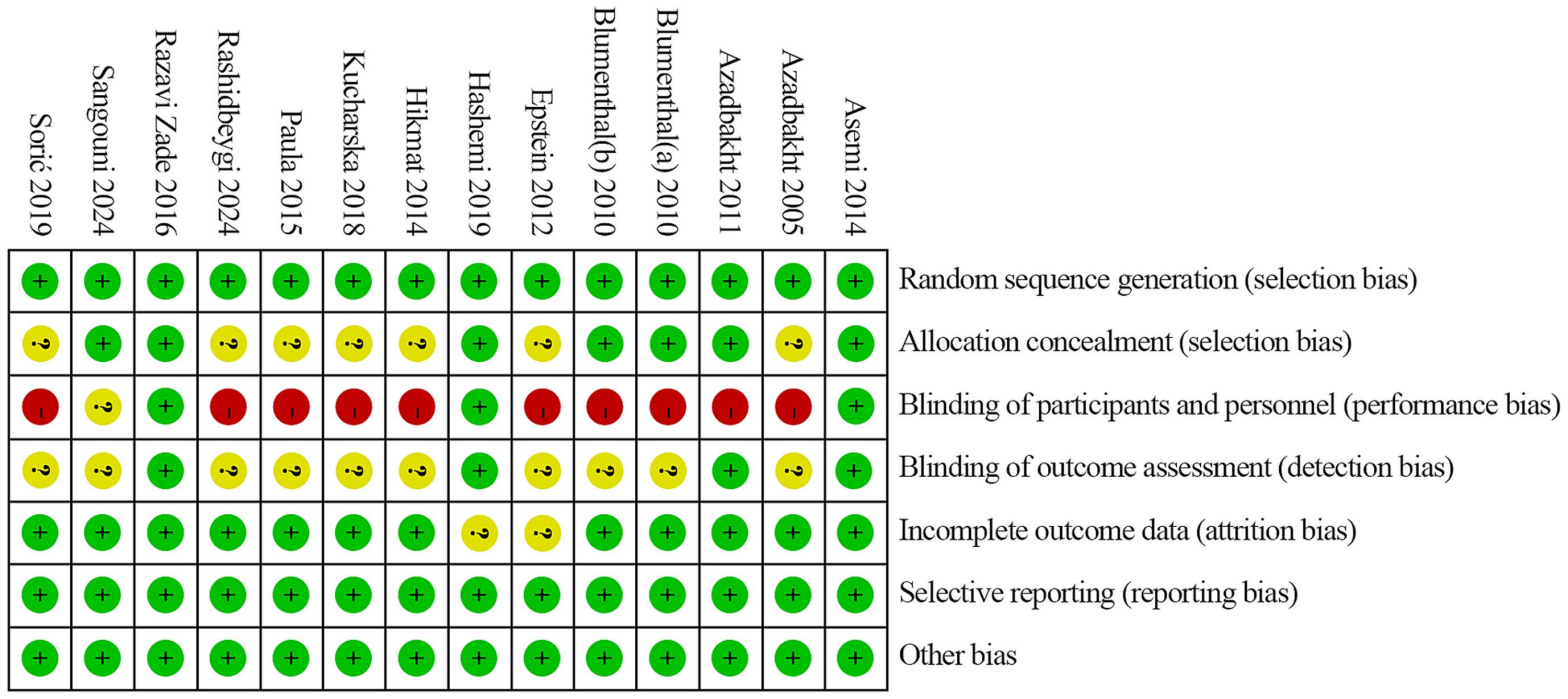
Risk of bias graph review authors’ judgments about each risk of bias item presented as percentages across all included studies.

We assessed study quality using the Cochrane risk-of-bias tool. All trials were rated as low risk for random sequence generation, suggesting that randomization was generally sound. However, information on allocation concealment was often insufficient, and only a minority of studies were clearly low risk in this domain. Most studies were rated as high risk for blinding of participants and study staff, indicating that group awareness could have influenced conduct or adherence. Blinding of outcome assessment was frequently unclear, reflecting limited reporting or inconsistent use of blinded assessment procedures. Most trials were rated as low risk for missing outcome data, and risks related to selective reporting or other potential sources of bias were generally low. Because dietary interventions are difficult to mask, the higher risk related to blinding is expected. Full assessments are shown in Figures 2, 3.

### 3.4 Results for the components of MetS

#### 3.4.1 WC

**Figure 4:**
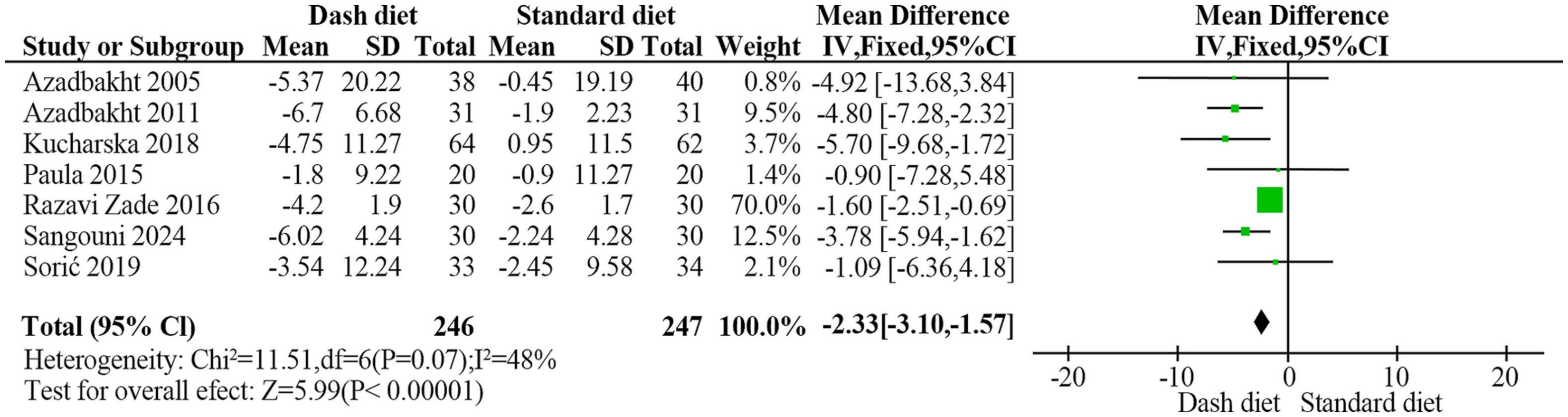
Forest plot of the effect of the DASH diet on waist circumference.

Seven RCTs (23, 24, 26–29, 35) involving 493 participants reported significant reductions in WC with the DASH diet compared to the non-DASH group (MD = −2.33 cm, 95% CI: −3.10 to −1.57, *p* < 0.001). Moderate heterogeneity was observed (*I*^2^ = 48%, *p* = 0.07) (Figure 4). The evidence quality was rated as moderate based on the GRADE assessment (Table 2).

**Table 2:** GRADE evidence profile.

Certainty assessment							№ of patients		Effect		Certainty	Importance
№ of studies	Study design	Risk of bias	Inconsistency	Indirectness	Imprecision	Other considerations	[Dash diet]	[Standard diet]	Relative (95% CI)	Absolute (95% CI)		
Waist circumference												
7	Randomized trials	Serious^a^	Not serious	Not serious	Not serious	None	246	247	–	−2.33 (−3.10 to −1.57)	⨁⨁⨁◯ Moderate	CRITICAL
Systolic blood pressure												
10	Randomized trials	Serious^a^	Not serious	Not serious	Not serious	None	380	386	–	−5.52 (−6.83 to −4.21)	⨁⨁⨁◯ Moderate^a^	CRITICAL
Diastolic blood pressure												
10	Randomized trials	Serious^a^	Not serious	Not serious	Not serious	None	380	386	–	−3.93 (−4.76 to −3.10)	⨁⨁⨁◯ Moderate^a^	CRITICAL
High-density lipoprotein cholesterol												
7	Randomized trials	Serious^a^	Serious^b^	Not serious	Not serious	None	214	218	–	1.44 (0.20 to 2.68)	⨁⨁◯◯ Low^a,b^	CRITICAL
Triglycerides												
8	Randomized trials	Serious^a^	Not serious	Not serious	Not serious	None	252	258	–	−16.57 (−23.53 to −9.61)	⨁⨁⨁◯ Moderate^a^	CRITICAL
Fasting blood glucose												
8	Randomized trials	Serious^a^	Serious^b^	Not serious	Not serious	None	312	314	–	−0.91 (−4.51 to 2.68)	⨁⨁◯◯ Low^a,b^	CRITICAL
HOMA-IR												
2	Randomized trials	Serious^a^	Not serious	Not serious	Not serious	None	94	92	–	− 0.71 (−1.10 to −0.31)	⨁⨁⨁◯ Moderate^a^	CRITICAL

CI, confidence interval; MD, mean difference. 1 Explanations. ^a^Blind method settings are suboptimal. ^b^Excessive heterogeneity.

#### 3.4.2 SBP

**Figure 5:**
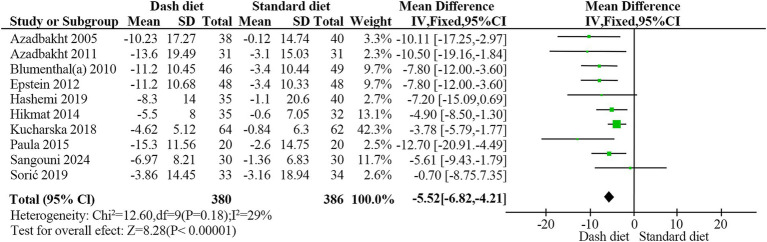
Forest plot of the effect of the DASH diet on systolic blood pressure.

Ten studies (23–26, 28, 29, 31, 34–36) with 766 participants showed that the DASH diet significantly reduced systolic blood pressure compared to the non-DASH group (MD = −5.52 mmHg, 95% CI: −6.83 to −4.21, *p* < 0.001). No significant heterogeneity was found (*I*^2^ = 29%, *p* = 0.18) (Figure 5). The evidence quality was rated as moderate based on the GRADE assessment (Table 2).

#### 3.4.3 DBP

**Figure 6:**
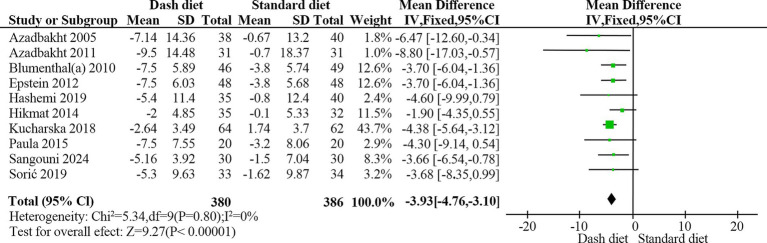
Forest plot of the effect of the DASH diet on diastolic blood pressure.

Ten RCTs (23–26, 28, 29, 31, 34–36) with 766 participants reported a significant reduction in diastolic blood pressure in the DASH group (MD = −3.93 mmHg, 95% CI: −4.76 to −3.10, *p* < 0.001). No heterogeneity was detected (*I*^2^ = 0%, *p* = 0.80) (Figure 6). The evidence quality was rated as moderate based on the GRADE assessment (Table 2).

#### 3.4.4 HDL-C

**Figure 7:**
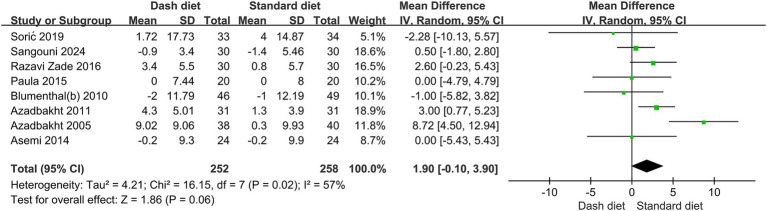
Forest plot of the effect of the DASH diet on high-density lipoprotein cholesterol.

Eight RCTs (23, 24, 27–29, 32, 33, 35) with 510 participants found no significant effect of the DASH diet on HDL-C levels (MD = 1.90 mg/dL, 95% CI: −0.10 to 3.90, *p* = 0.06). Significant heterogeneity was observed (*I*^2^ = 57%, *p* = 0.02) (Figure 7). The evidence quality was low based on the GRADE assessment (Table 2).

**Figure 8:**
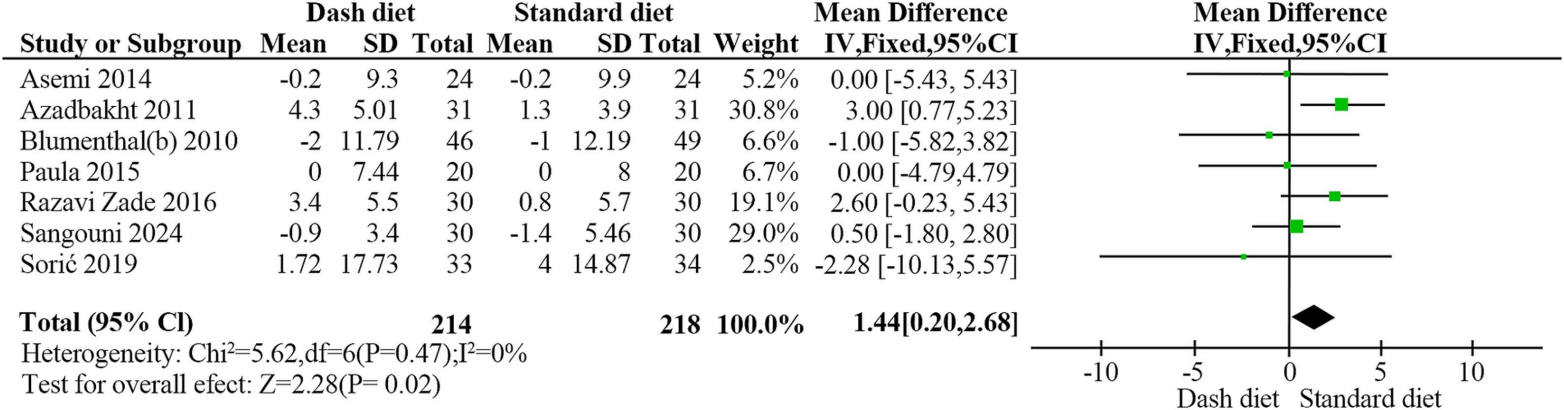
Forest plot of the effect of the DASH diet on high-density lipoprotein cholesterol(after exclusion).

However, sensitivity analyses identified one study as a major contributor to heterogeneity, and its inclusion materially changed the statistical significance of the pooled results. After excluding one study (35), identified as a major source of heterogeneity, the remaining 432 participants (214 in the DASH group and 218 in the non-DASH group) showed a significant increase in HDL-C levels with the DASH diet (MD = 1.44 mg/dL, 95% CI: 0.20–2.68, *p* = 0.02). No heterogeneity was observed (*I*^2^ = 0%, *p* = 0.47) (Figure 8).

#### 3.4.5 TG

**Figure 9:**
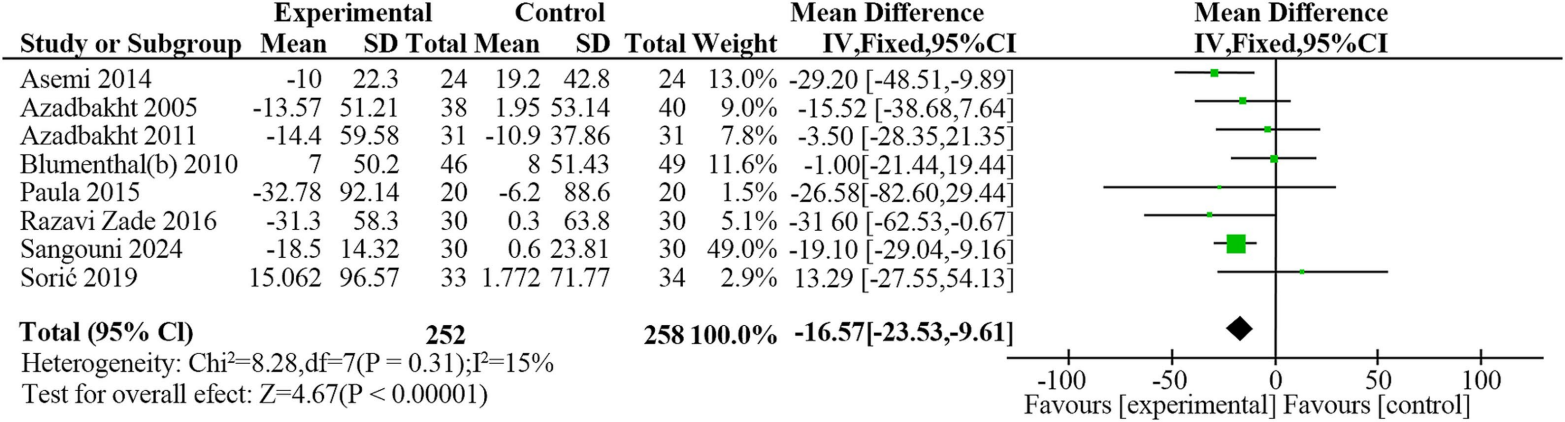
Forest plot of the effect of the DASH diet on triglycerides.

Eight studies (23, 24, 27–29, 32, 33, 35) reported TGlevels in 510 participants. The DASH diet significantly reduced TG levels compared to the non-DASH group (MD = −16.57 mg/dL, 95% CI: −23.53 to −9.61, *p* < 0.001), with minimal heterogeneity (*I*^2^ = 15.4%, *p* = 0.31) (Figure 9). The evidence quality was moderate based on the GRADE assessment (Table 2).

#### 3.4.6 FBG

**Figure 10:**
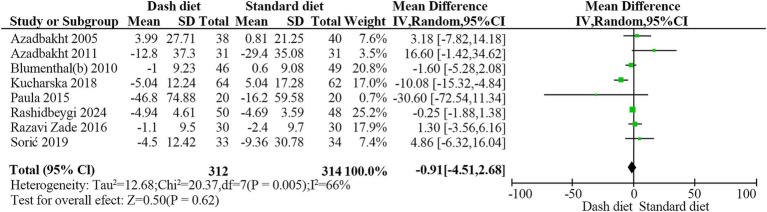
Forest plot showing the effect of the DASH diet on fasting blood glucose levels.

Eight trials (24, 26–30, 33, 35) with 626 participants found no significant effect of the DASH diet on FBG levels (MD = −0.91 mg/dL, 95% CI: −4.51 to 2.68, *p* = 0.62). Significant heterogeneity was observed (*I*^2^ = 66%, *p* = 0.005) (Figure 10). The evidence quality was rated as low based on the GRADE assessment (Table 2).

#### 3.4.7 HOMA-IR

**Figure 11:**
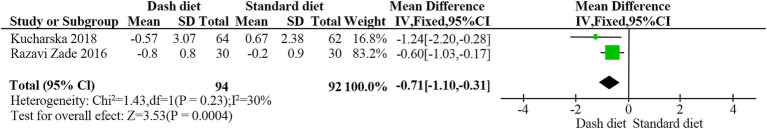
Forest plot of the effect of the DASH diet on HOMA-IR.

Two RCTs (26, 27) involving 186 participants assessed HOMA-IR levels, showing a significant reduction in the DASH group compared to the non-DASH group (MD = −0.71, 95% CI: −1.10 to −0.31, *p* < 0.001). No significant heterogeneity was observed (*I*^2^ = 29.9%, *p* = 0.23) (Figure 11). The evidence quality was moderate based on the GRADE assessment (Table 2).

### 3.5 Subgroup analysis and sensitivity analysis

We conducted subgroup analyses for outcomes with sufficient data (WC, HDL-C, FBG, and BP) to assess whether intervention effects differed across clinical subgroups (Tables 3, 4). Given that central obesity is a core diagnostic criterion for MetS and a key component of the condition, a primary objective of these subgroup analyses was to examine whether intervention duration and participants’ age modified the improvement in central obesity. WC was used as a surrogate measure of central obesity. We found that reductions in WC were greater when the intervention lasted longer than 12 weeks, whereas no clear differences in WC improvement were observed between participants aged ≥50 years and those aged <50 years. Collectively, these findings suggest that a longer intervention duration may be associated with greater improvements in central obesity, largely independent of age. Additionally, our subgroup analyses suggest that a 12-week DASH dietary intervention may be sufficient to produce measurable improvements in key features of metabolic syndrome, including BP, WC, and TG levels.

**Table 3:** Subgroup analysis by age.

Subgroup	No. of trials	Mean	95% CI	*p*	*I*^2^(%)	*p*-value of heterogeneity	Between-group *p-*values
WC							
Overall	6	−2.07	−2.88, −1.72	<0.001	32	0.020	0.34
Age subgroup							
≥50 years	3	−3.41	−6.25, −0.56	0.02	23	0.27	
<50 years	3	−1.96	−2.79, −1.12	<0.001	47	0.15	
HDL-C							
Overall	6	0.75	−0.74,2.24	0.32	0	0.71	0.274
Age subgroup							
≥50 years	3	−0.78	−3.90,2.34	0.62	0	0.88	
<50 years	3	1.20	−0.49,2.90	0.16	0	0.48	
FBG							
Overall	7	−1.55	−4.97,1.87	0.37	64	0.01	0.30
Age subgroup							
≥50 years	4	−4.07	−11.50,3.36	0.28	72	0.01	
<50 years	3	−0.03	−1.56,1.50	0.97	0	0.71	
SBP							
Overall	8	−5.14	−6.53, −3.75	<0.001	29	0.19	0.411
Age subgroup							
≥50 years	4	−4.71	−6.44, −2.98	<0.001	60	0.06	
<50 years	4	−5.94	−8.29, −3.59	0.001	0	0.62	
DBP							
Overall	8	−3.90	−4.80, −3.01	<0.001	0	0.79	0.289
Age subgroup							
≥50 years	4	−4.20	−5.26, −3.15	<0.001	0	0.96	
<50 years	4	−3.12	−4.82, −1.43	<0.001	0	0.47	
TG							
Overall	6	−17.92	−25.56, −10.29	0	29	0.21	0.037
Age subgroup							
≥50 years	3	−0.87	−18.25,16.50	0.92	0	0.53	
<50 years	3	−22.00	−30.50, −13.50	0.001	0	0.54	

**Table 4:** Subgroup analysis by intervention duration.

Subgroup	No. of trials	Mean	95% CI	*p*	*I*^2^(%)	*p-*value of heterogeneity	Between-group *p*-values
WC							
Overall	7	−2.33	−3.10, −1.57	<0.001	47.9	0.074	0.048
Study duration							
≥12 weeks	4	−3.90	−5.65, −2.15	<0.001	0	0.585	
<12 weeks	3	−1.96	−2.81, −1.11	<0.001	65.2	0.057	
HDL-C							
Overall	7	1.44	0.20,2.68	0.023	0	0.467	0.091
Study duration							
≥12 weeks	3	0.06	−1.95,2.07	0.955	78.4	0.003	
<12 weeks	4	2.30	0.72,3.88	0.004	0	0.574	
FBG							
Overall	8	−0.91	−4.51,2.68	0.619	65.6	0.005	0.58
Study duration							
≥12 weeks	5	−1.93	−5.99,2.14	0.353	71.5	0.007	
<12 weeks	3	2.73	−13.11,18.57	0.736	59.7	0.085	
SBP							
Overall	10	−5.52	−6.83, −4.21	<0.001	28.6	0.181	0.40
Study duration							
≥12 weeks	7	−5.26	−6.70, −3.82	<0.001	26	0.23	
<12 weeks	3	−6.71	−9.79, −3.63	0.001	47	0.15	
DBP							
Overall	10	−3.93	−4.76, −3.10	<0.001	0	0.804	0.26
Study duration							
≥12 weeks	7	−4.13	−5.04, −3.23	<0.001	0	0.98	
<12 weeks	3	−2.81	−4.92, −0.70	0.0009	32	0.23	
TG							
Overall	8	−16.57	−23.53, −9.61	<0.001	15.4	0.309	0.334
Study duration							
≥12 weeks	4	−14.46	−22.64, −6.29	0.001	30.0	0.232	
<12 weeks	4	−22.15	−35.44, −8.87	0.001	1.9	0.383	

Sensitivity analyses showed that the results were robust for all outcomes, except for HDL-C, which exhibited some variation after exclusion of one study (35). For the remaining outcomes, leave-one-out analyses did not change the direction of the pooled effect estimates, and the overall conclusions remained consistent. The detailed findings for HDL-C before and after study exclusion are described in the preceding section.

### 3.6 Publication bias

Upon visual inspection of the funnel plot (Supplementary Figures 1–7), a slight asymmetry was observed, which was likely due to the limited number of studies included. Egger’s test showed no clear evidence of publication bias for most outcomes (all *p* > 0.05). The only exception was systolic blood pressure (SBP) (*p* = 0.036), so further assessment was limited to SBP using the trim-and-fill approach.

Before adjustment, the fixed-effect meta-analysis indicated that the intervention significantly reduced SBP (MD = −5.52 mmHg, 95% CI −6.83 to −4.21; *p* < 0.001). After reanalysis using the trim-and-fill method, the effect size was slightly reduced but remained statistically significant (MD = −5.05 mmHg, 95% CI −5.90 to −4.20; *p* < 0.001). Taken together, the results were stable after adjustment, supporting the robustness of the observed SBP reduction.

### 3.7 GRADE certainty of evidence

The GRADE evidence profiles are shown in Table 2. With the exception of FBG and HDL-C, which were rated as low certainty, all other outcomes were rated as moderate certainty.

## 4 Discussion

### 4.1 Main findings

This systematic review and meta-analysis included 14 RCTs involving 1,062 people with MetS. Across studies, the DASH diet was associated with overall improvements in several key components of MetS compared with control diets, including measures linked to central obesity, BP, TG, and insulin resistance. In contrast, the overall effects on FBG were not statistically significant. Notably, the pooled HDL-C result was sensitive to a single study, suggesting that the effect may depend on differences in study methods, intervention delivery, or participant characteristics.

Subgroup analyses further suggested that longer intervention duration was associated with greater improvements in central adiposity, largely independent of age strata. In terms of evidence certainty, FBG and HDL-C outcomes were supported by low-certainty evidence, whereas most other outcomes reached moderate certainty. Sensitivity analyses generally corroborated the robustness of the main findings, and the direction of the SBP effect remained consistent after adjustment for potential publication bias.

### 4.2 Comparison with existing literature

To our knowledge, this study is the first meta-analysis to specifically evaluate the effect of the DASH diet on all components of MetS. Previous meta-analyses in this field have mainly investigated the effects of the DASH diet on only some components of MetS (18), lacking a comprehensive assessment of all components, and thus provided limited guidance for clinical practice. To offer a more complete and current evidence base, we included recently published RCTs and evaluated the DASH diet’s impact on all MetS components: WC, BP, HDL-C, TG, FBG, and HOMA-IR.

With respect to WC and HOMA-IR, our findings are broadly consistent with the existing literature and further support the potential benefits of the DASH dietary pattern for metabolic phenotypes related to metabolic syndrome (MetS) (37, 38), with particularly prominent improvements in central adiposity and insulin resistance. Central obesity is not only a defining and arguably the most pivotal component of MetS (39), but is also tightly linked to visceral fat accumulation, which can exacerbate insulin resistance through increased free fatty acid spillover, lipotoxicity, and chronic low-grade inflammation. Insulin resistance, in turn, is a unifying feature across the MetS spectrum, driving downstream disturbances in glucose and lipid metabolism and ultimately heightening cardiovascular risk (40, 41). Accordingly, the observed reductions in WC alongside improvements in HOMA-IR in our study suggest that adherence to the DASH diet may act on key pathogenic pathways of MetS, thereby conferring broader, favorable effects on overall metabolic risk (23).

In terms of BP, our analysis confirms previous studies that found significant reductions in blood pressure among individuals following the DASH diet (14, 15, 18, 42–44). This effect is plausibly attributable to the diet’s defining features—lower sodium intake, higher potassium intake, greater dietary fiber, and an abundance of antioxidant nutrients such as magnesium and vitamin C (14). The INTERSALT study further corroborated potassium as a key determinant of blood pressure (45). Moreover, a meta-analysis by Binia and colleagues demonstrated that potassium supplementation lowers BP in both hypertensive and normotensive individuals (46). Notably, magnesium deficiency is common among patients with metabolic syndrome and has been linked to an increased risk of cardiovascular disease; therefore, the magnesium-rich composition of the DASH diet may be particularly advantageous in this population (47, 48).

In addition to lowering BP, our study found that the DASH diet reduced TG concentrations in patients with MetS. After excluding one highly heterogeneous study, sensitivity analyses further suggested that, compared with a standard diet, the DASH diet increased HDL-C. These findings align with recent studies and indicate that the DASH dietary pattern may improve lipid profiles, which could help reduce the elevated cardiovascular risk associated with MetS (13, 49, 50). Elevated TG levels are associated with an increased risk of cardiovascular diseases, while HDL-C plays a protective role by facilitating cholesterol transport to the liver for metabolism, thus reducing the risk of atherosclerosis and coronary artery disease (51–53). Thus, the potential benefits of the DASH diet in reducing TG and increasing high-density lipoprotein cholesterol may represent a key pathway through which it improves cardiovascular risk in patients with metabolic syndrome. This further supports the clinical value of dietary interventions, including the DASH diet, in the management of metabolic disorders.

Although we observed a negative trend between the DASH diet and FBG levels, this relationship was not statistically significant. Similar results were reported by Shirani and Siervo (37, 54). By contrast, studies by Sun et al. and Shirani et al. have indicated that the DASH diet has a significant glucose-lowering effect (37, 55). Taken together, these discrepant results imply that, in certain populations or under specific study conditions, any improvement in FBG attributable to the DASH diet may be present but modest in magnitude, and therefore readily attenuated by differences in study design and population heterogeneity. Differences in baseline metabolic health may partly explain this variation. In people with metabolic syndrome, fasting glucose is strongly influenced by excess glucose production by the liver, which is less responsive to insulin, and this is often accompanied by abdominal fat and fatty liver (40). As a result, improving diet quality alone may not lead to a clear short-term reduction in FBG. In addition, medication use and lifestyle behaviors differ widely in this group, and fasting glucose can fluctuate with recent food intake, sleep, and stress, increasing variability and making effects harder to detect (56, 57). Therefore, benefits of the DASH diet may be more readily captured by measures of insulin resistance (e.g., HOMA-IR) or longer-term glycemic markers such as HbA1c rather than by FBG alone.

### 4.3 Implications for clinical practice

The DASH diet is easy to adopt, grounded in natural foods, and does not require complex calculations or specialized products, making it highly sustainable for long-term use (58). Compared to pharmacological treatments, it offers a safe and natural alternative that promotes metabolic health. While medications may be associated with side effects, the DASH diet enhances overall dietary habits, effectively reducing the risk of diseases linked to MetS, such as hypertension, diabetes, and cardiovascular disease. Therefore, the DASH diet should be recommended as the first-line dietary intervention for patients with metabolic syndrome, as it can produce significant benefits within 12 weeks without the need for pharmacological intervention.

### 4.4 Strengths and limitations

This meta-analysis offers several notable strengths. Our systematic review and meta-analysis comprehensively investigate the relationship between the DASH diet and MetS components, using established diagnostic criteria. We emphasize the beneficial effects of the DASH diet on these components. To our knowledge, this is the first meta-analysis to assess the impact of the DASH diet on MetS and its individual components. Compared to previous meta-analyses (18, 59), this study provides a systematic and comprehensive synthesis of the DASH diet’s multidimensional effects. The analysis adhered to current reporting guidelines, including the PRISMA statement, and was registered with PROSPERO. We employed sensitivity analyses, funnel plots to assess publication bias, Egger’s test for statistical significance, and the GRADE methodology to evaluate evidence certainty. Furthermore, all included studies were RCTs, ensuring high-quality and representative findings.

However, several limitations should be considered. The included studies varied widely in terms of intervention duration, and most trials did not report or quantitatively assess adherence to the DASH diet. Moreover, there may have been differences across studies in the definitions and measurement methods of certain outcome parameters, including blood pressure, waist circumference, and HOMA-IR, which may have contributed to between-study heterogeneity. Residual confounding factors also could not be ruled out. Additionally, the sample sizes were relatively small. Therefore, larger studies with longer follow-up periods and standardized, quantitative assessments of dietary adherence and outcome measures are needed to further elucidate the role of the DASH diet in managing MetS.

## 5 Conclusion

This study demonstrates that the DASH diet significantly improves several key components of MetS, particularly with clear effects on WC, BP, TG, and HDL-C. Although its direct effect on FBG is limited, the diet shows promise in improving insulin resistance. Based on these findings, we propose two key insights: 1. The DASH diet can serve as a first-line non-pharmacological intervention for MetS; 2. To further enhance its clinical application, long-term, large-scale intervention studies are required, especially to assess its applicability and feasibility across diverse populations and cultural contexts.

## Data Availability

The original contributions presented in the study are included in the article/Supplementary material, further inquiries can be directed to the corresponding author.
