# Supplementary material for: The effect of DASH diet on components of metabolic syndrome: a systematic review and meta-analysis of randomized controlled trials
Source: Front Nutr. 2026 May 11;13:1738410. doi: 10.3389/fnut.2026.1738410 (PMC13199047; doi:10.3389/fnut.2026.1738410)
Supplement: Supplementary file 1 [file Table_1.docx]

**Supplementary Appendix**

**Table S1.** Search strategy

| **#** | **Searches** |
| --- | --- |
| **1** | Dietary Approaches to Stop Hypertension"[Title/Abstract] OR DASH[Title/Abstract] OR "DASH diet"[Title/Abstract] OR "Dietary Approaches to Stop Hypertension"[MeSH Terms] |
| **2** | " Metabolic Syndrome"[MeSH Terms] OR "metabolic syndrome"[Title/Abstract] OR "syndrome X"[Title/Abstract] OR "insulin resistance"[MeSH Terms] OR "insulin resistance"[Title/Abstract] OR "blood pressure"[MeSH Terms] OR "blood pressure"[Title/Abstract] OR hypertension[MeSH Terms] OR hypertension[Title/Abstract] OR "waist circumference"[MeSH Terms] OR "waist circumference"[Title/Abstract] OR obesity[MeSH Terms] OR obesity[Title/Abstract] OR overweight[MeSH Terms] OR overweight[Title/Abstract] OR "body weight"[MeSH Terms] OR "body weight"[Title/Abstract] OR BMI[Title/Abstract] OR "body mass index"[Title/Abstract] OR dyslipidemias[MeSH Terms] OR dyslipidemia*[Title/Abstract] OR triglycerides[MeSH Terms] OR triglyceride*[Title/Abstract] OR TG[Title/Abstract] OR "HDL"[Title/Abstract] OR "high density lipoprotein"[Title/Abstract] OR "cholesterol, HDL"[MeSH Terms] OR "LDL"[Title/Abstract] OR "low density lipoprotein"[Title/Abstract] OR "cholesterol, LDL"[MeSH Terms] OR cholesterol[MeSH Terms] OR cholesterol[Title/Abstract] OR "total cholesterol"[Title/Abstract] OR "blood glucose"[MeSH Terms] OR glucose[Title/Abstract] OR "fasting glucose"[Title/Abstract] OR insulin[MeSH Terms] OR insulin[Title/Abstract] OR HOMA[Title/Abstract] OR "HOMA-IR"[Title/Abstract] |
| **3** | randomized controlled trial[Publication Type] OR controlled clinical trial[Publication Type] OR random*[Title/Abstract] OR randomized[Title/Abstract] OR placebo[Title/Abstract] OR "clinical trial"[Title/Abstract] OR trial[Title/Abstract] OR "random allocation"[MeSH Terms] OR "double-blind method"[MeSH Terms] OR "single-blind method"[MeSH Terms] |
| **4** | #1 AND #2 AND #3 |


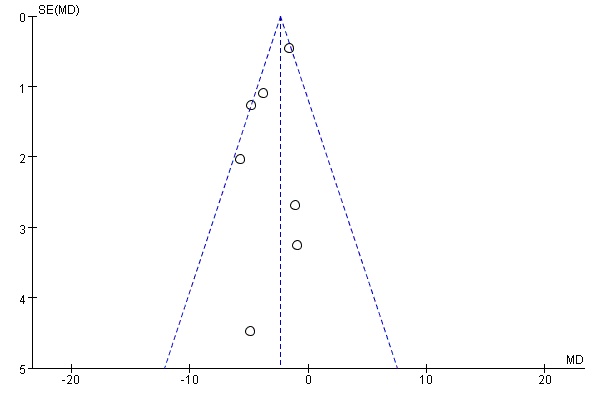


**Supplementary Figure 1.** Funnel plot of the effect of the DASH diet on waist circumference.


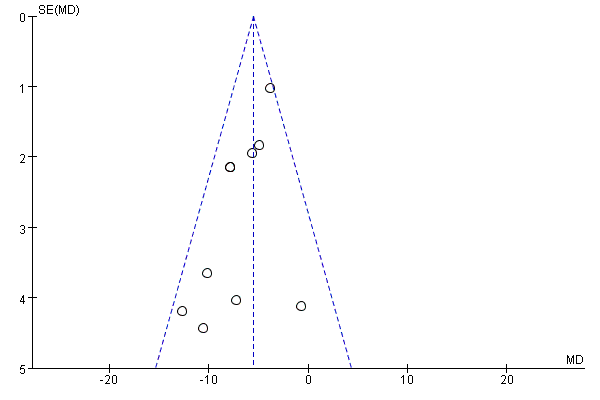


**Supplementary Figure 2.** Funnel plot of the effect of the DASH diet on systolic blood pressure.


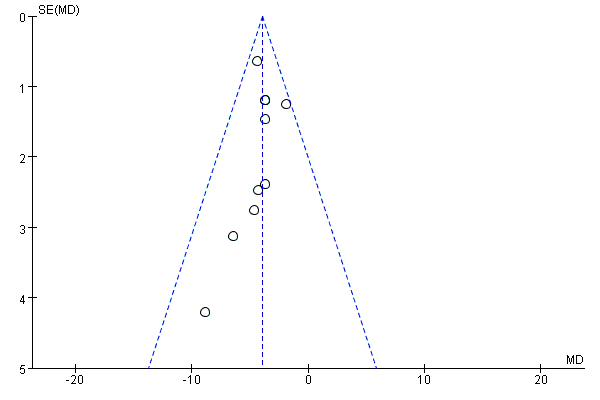


**Supplementary Figure 3.** Funnel plot of the effect of the DASH diet on diastolic blood pressure.


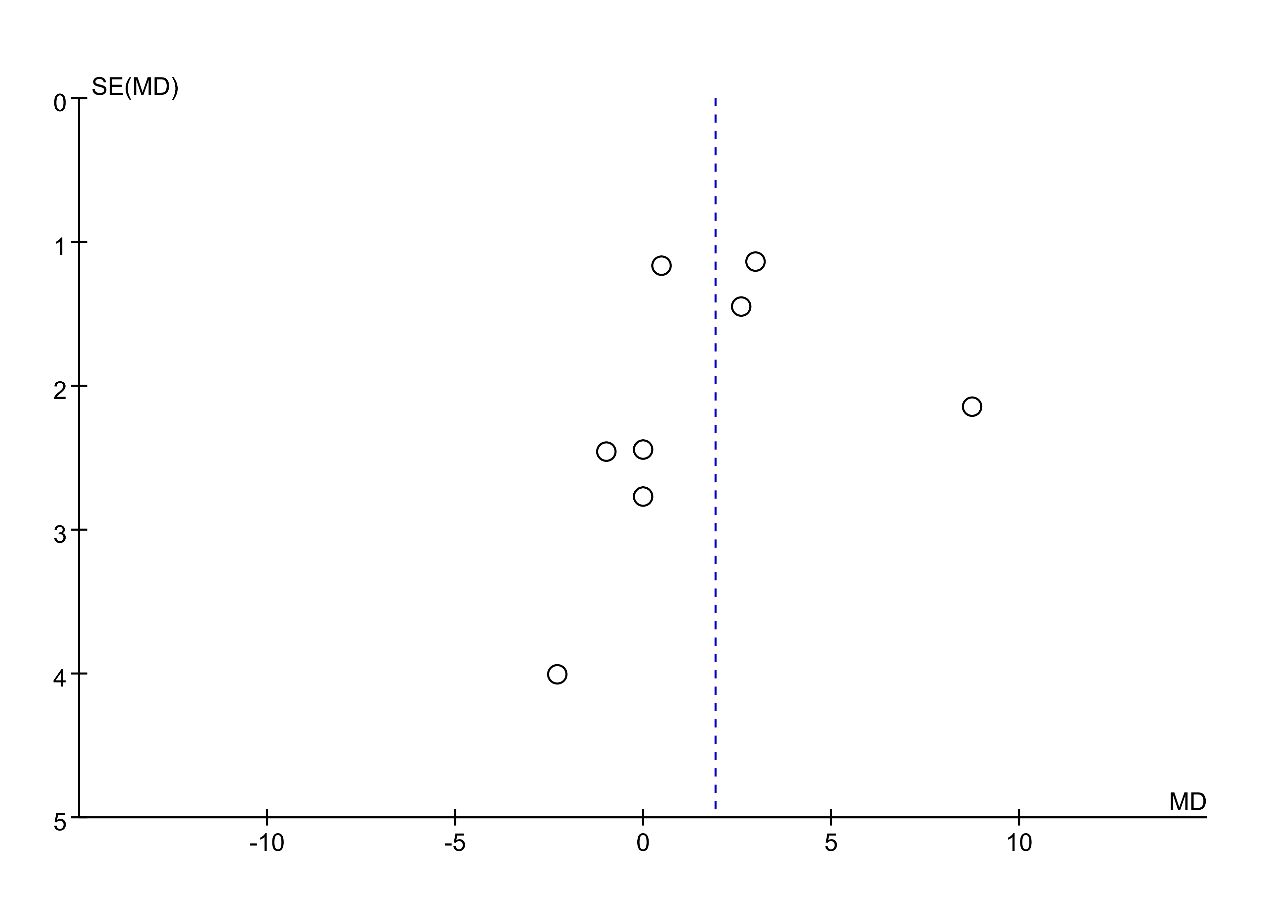


**Supplementary Figure 4(a).** Funnel plot of the effect of the DASH diet on high-density lipoprotein cholesterol.


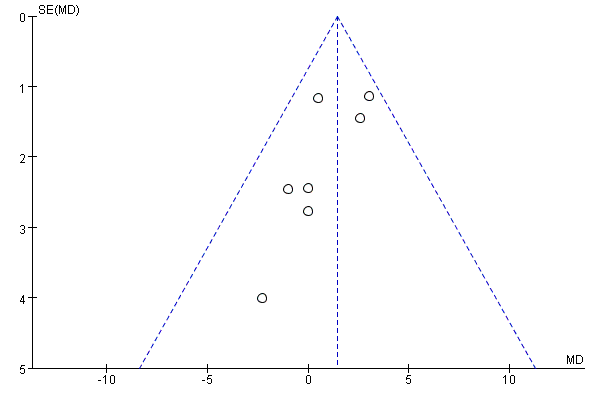


**Supplementary Figure 4(b).** Funnel plot of the effect of the DASH diet on high-density lipoprotein cholesterol.


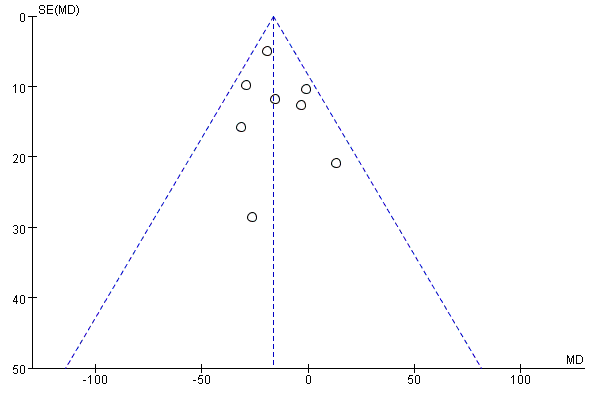


**Supplementary Figure 5.** Funnel plot showing the effect of the DASH diet on triglycerides.


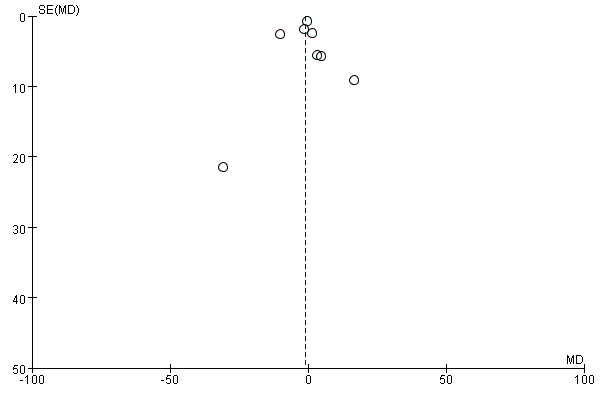


**Supplementary Figure 6.** Funnel plot of the effect of the DASH diet on fasting blood glucose levels.


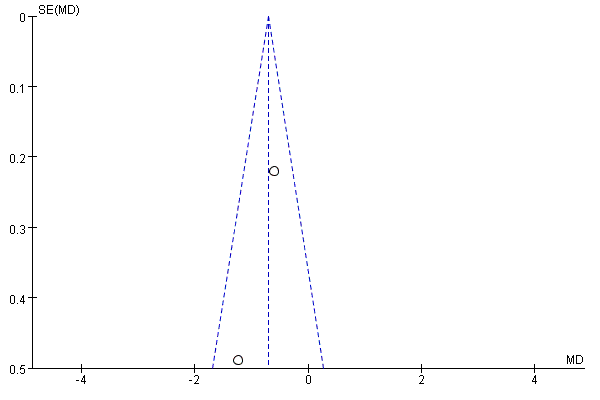


**Supplementary Figure 7.** Funnel plot of the effect of the DASH diet on HOMA-IR.


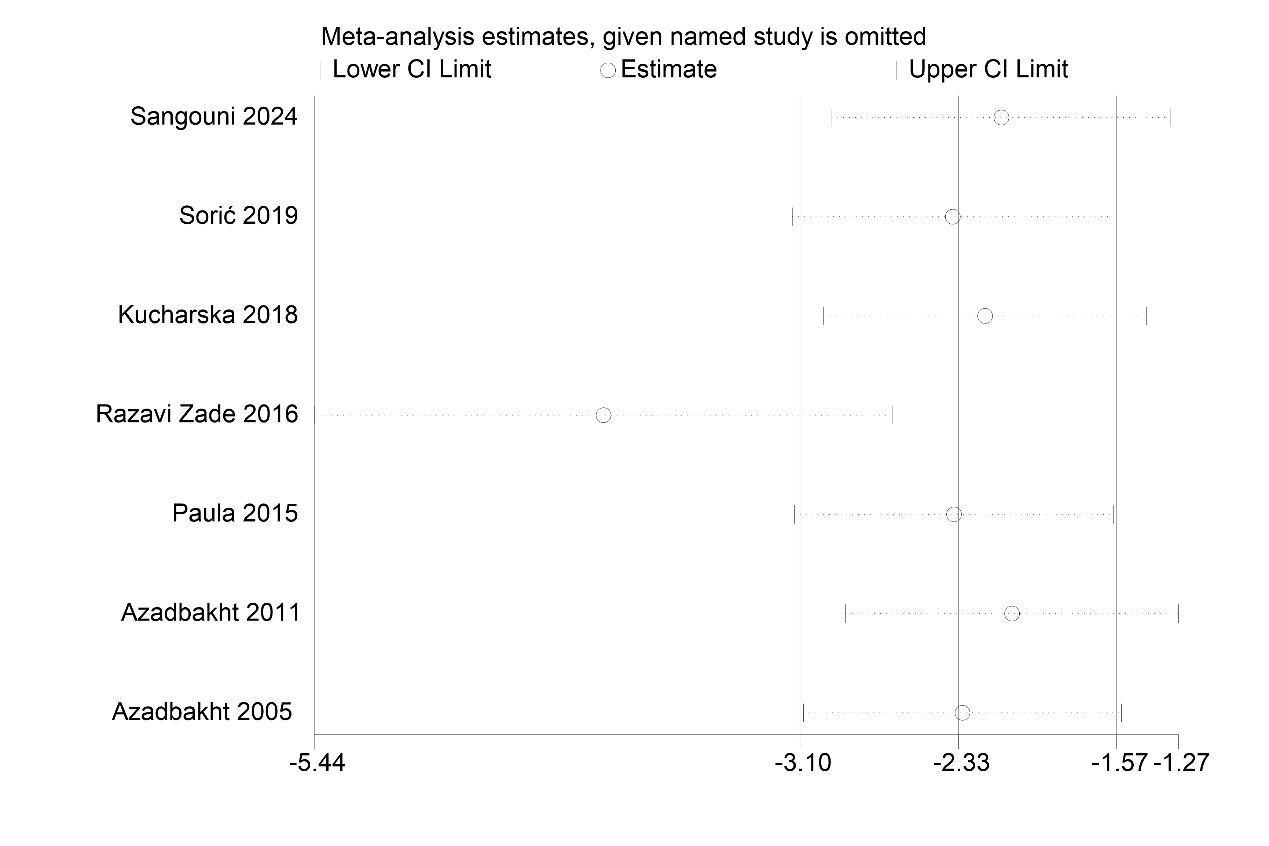


**Supplementary Figure 8.** Sensitivity analysis of the DASH diet's effect on waist circumference.
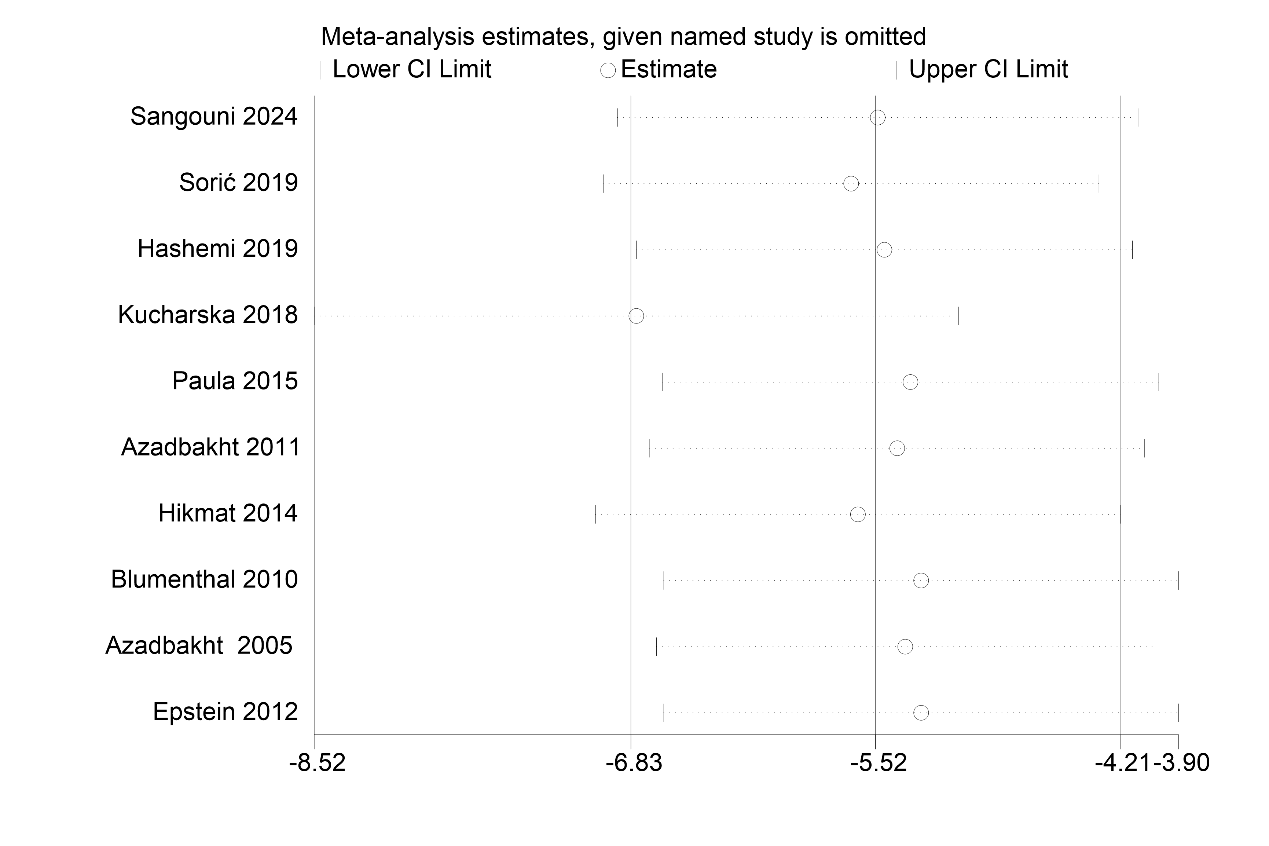


**Supplementary Figure 9.** Sensitivity analysis of the DASH diet's effect on systolic blood pressure.


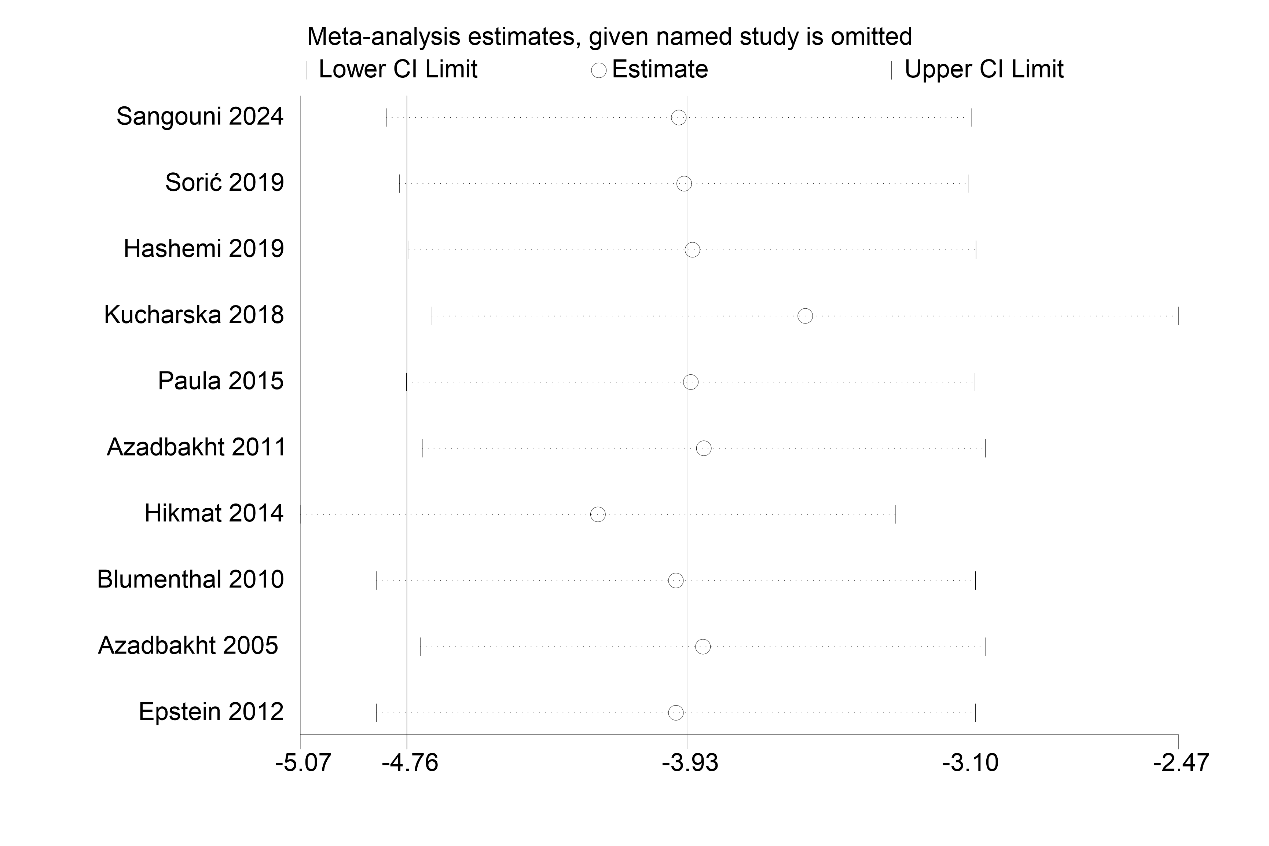


**Supplementary Figure 10.** Sensitivity analysis of the DASH diet's effect on diastolic blood pressure.


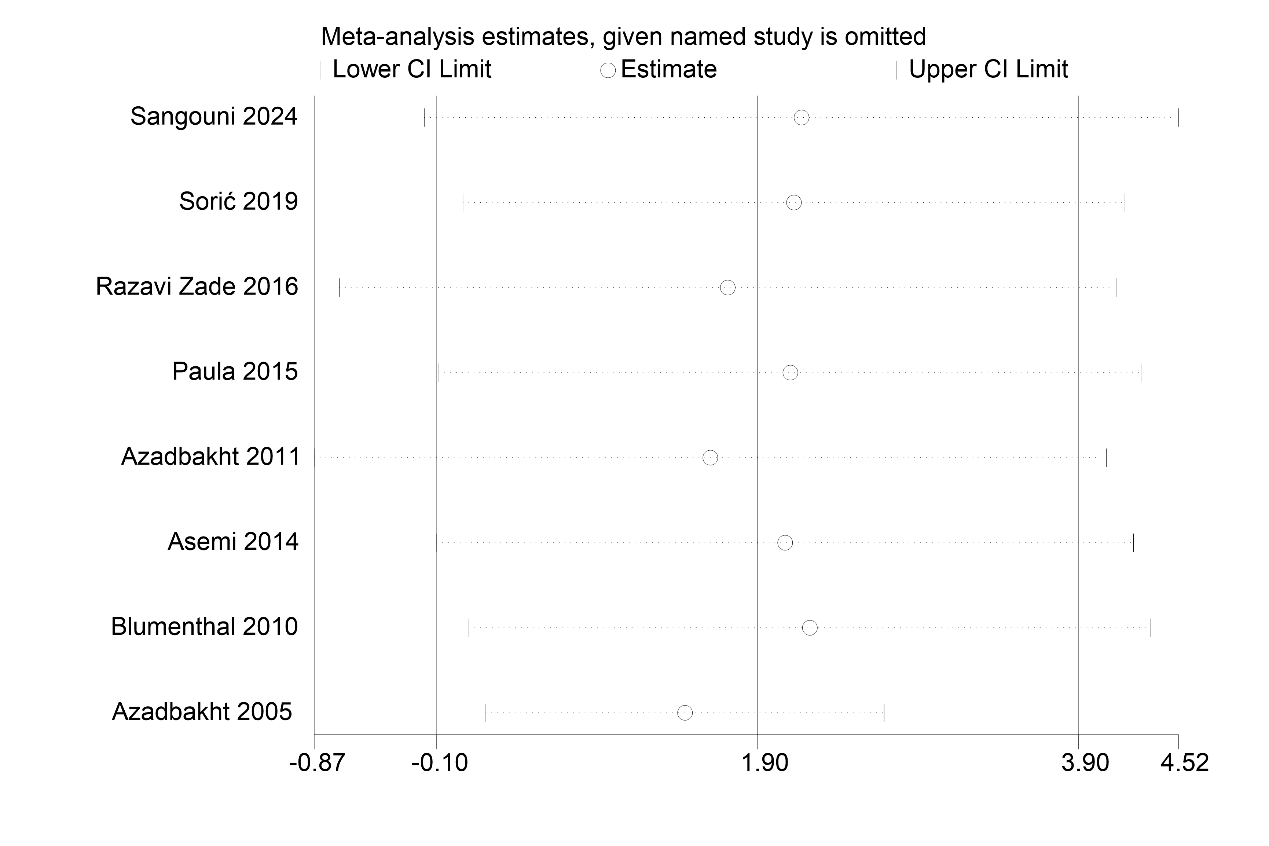


**Supplementary Figure 11.** Sensitivity analysis of the DASH diet's effect on high-density lipoprotein cholesterol.


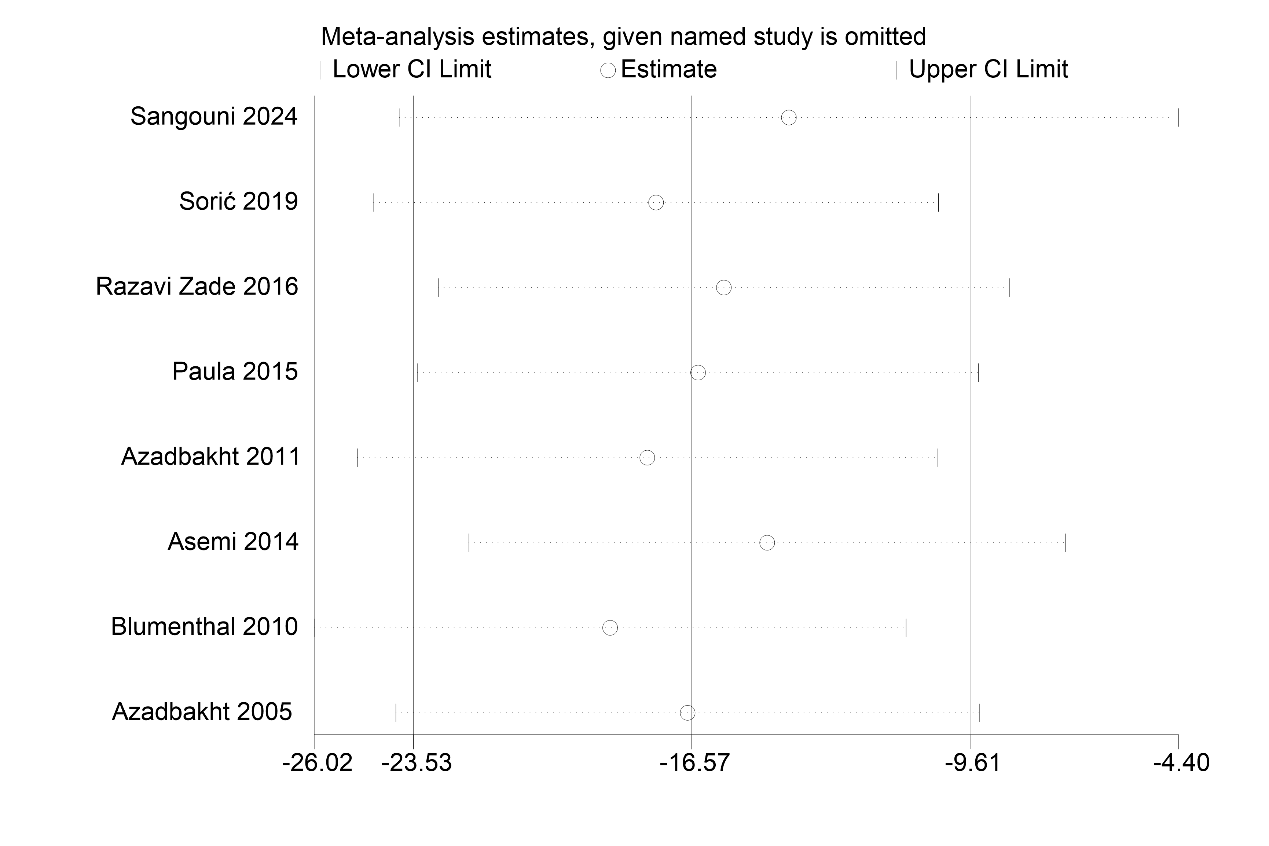


**Supplementary Figure 12.** Sensitivity analysis of the DASH diet's effect on triglycerides.


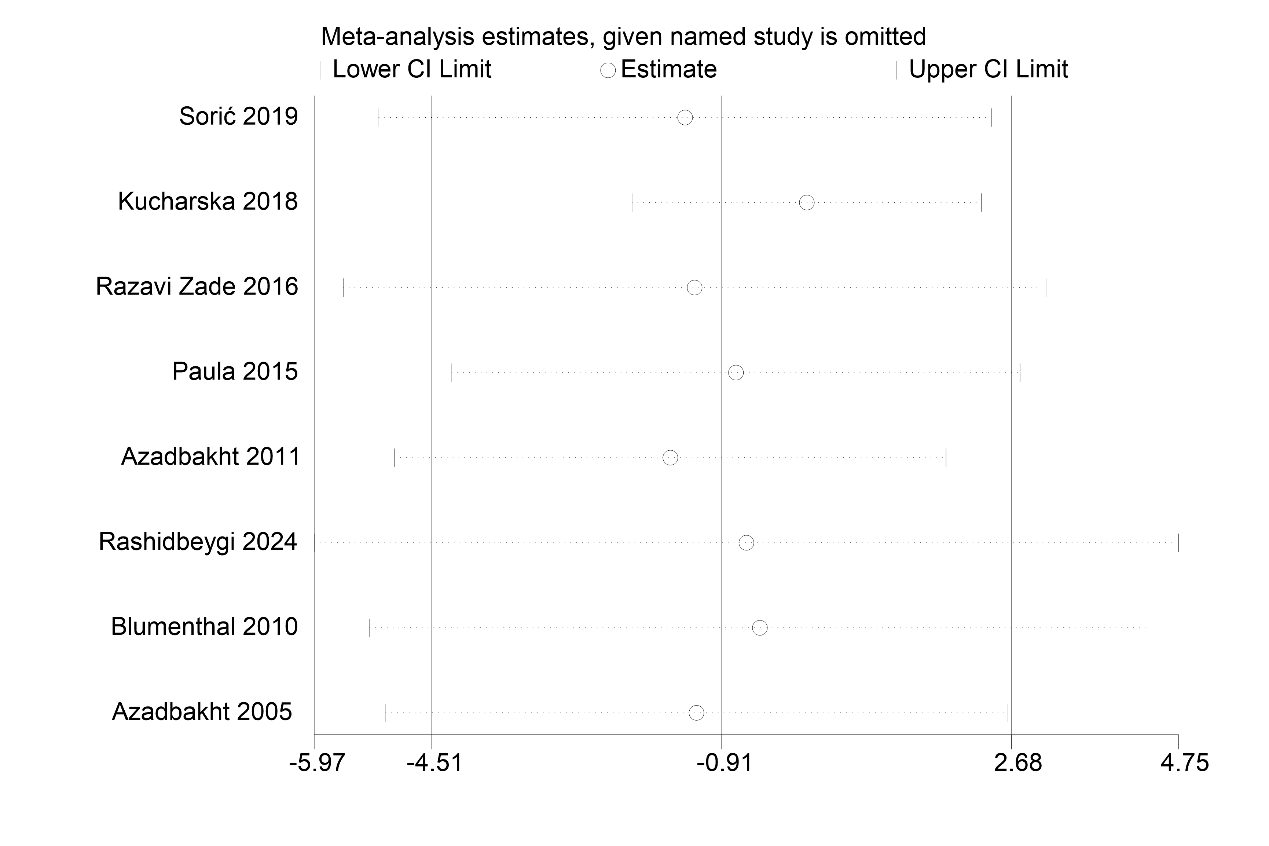


**Supplementary Figure 13.**Sensitivity analysis of the DASH diet's effect on fasting blood glucose levels.


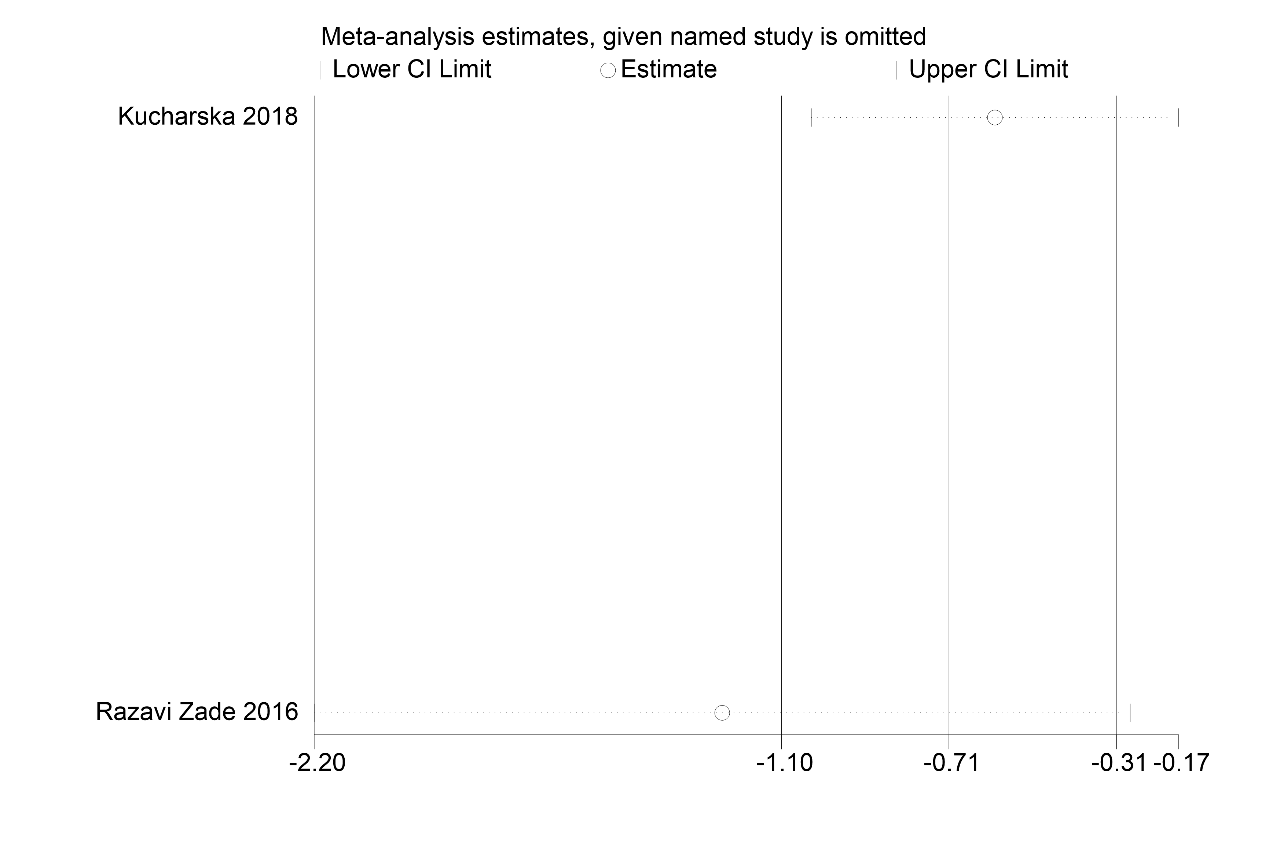


**Supplementary Figure 14.**Sensitivity analysis of the effect of the DASH diet on HOMA-IR.


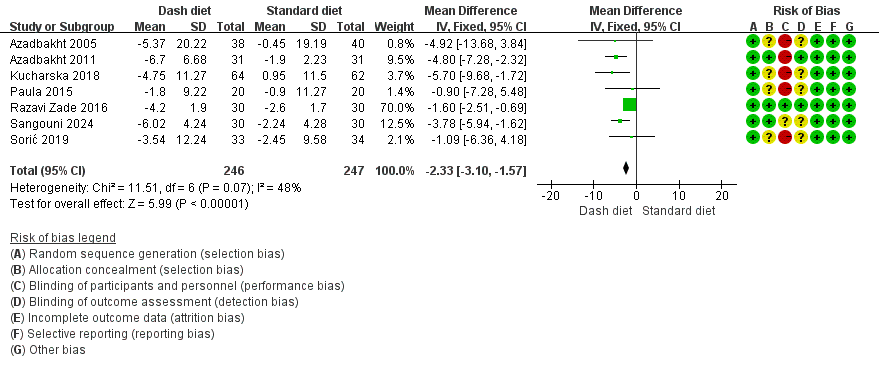


**Supplementary Figure 15.** Forest plot and bias risk for waist circumference.


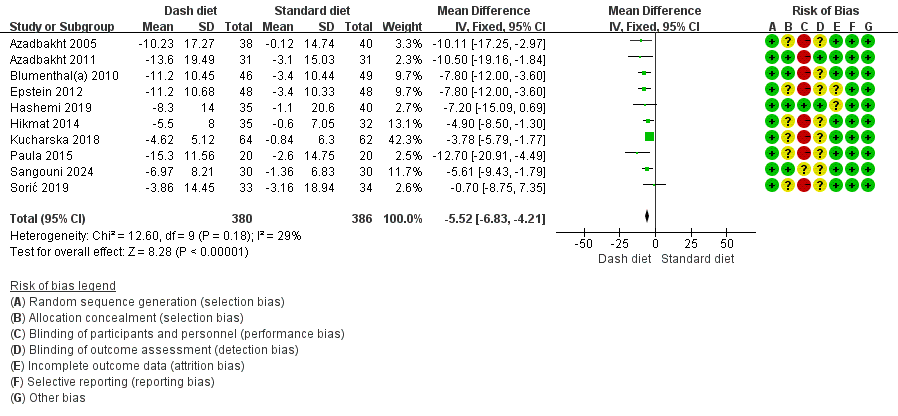


**Supplementary Figure 16.** Forest plot and bias risk for systolic blood pressure.


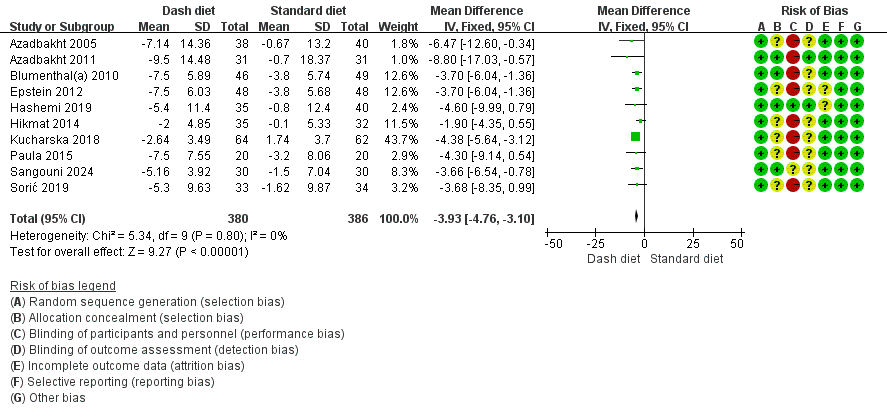


**Supplementary Figure 17.** Forest plot and risk of bias for diastolic blood pressure.


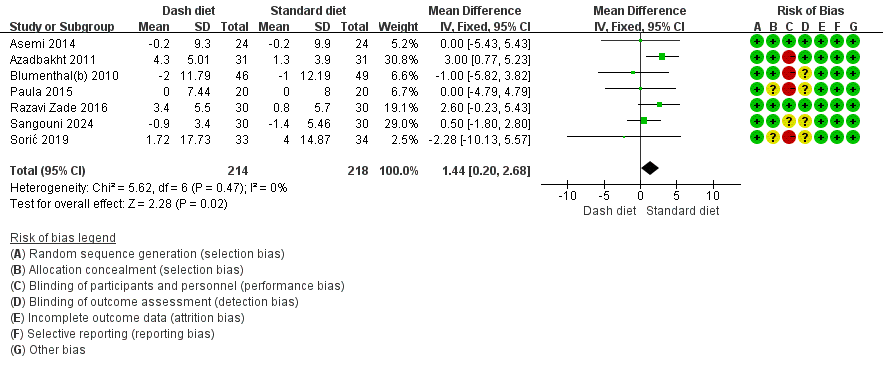


**Supplementary Figure 18.** Forest plot and bias risk for high-density lipoprotein cholesterol.


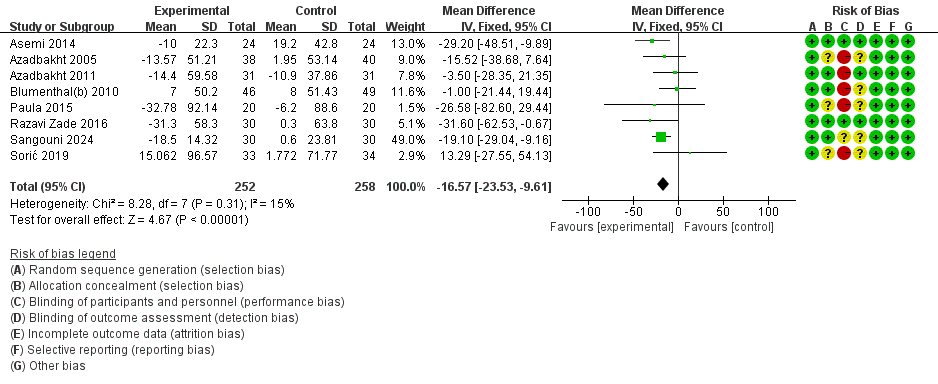


**Supplementary Figure 19.** Forest plot and bias risk for triglycerides.


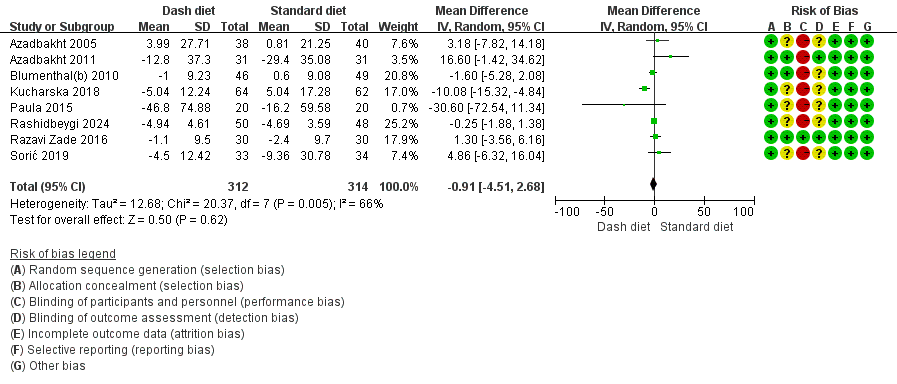


**Supplementary Figure 20.** Forest plot and bias risk for fasting blood glucose.


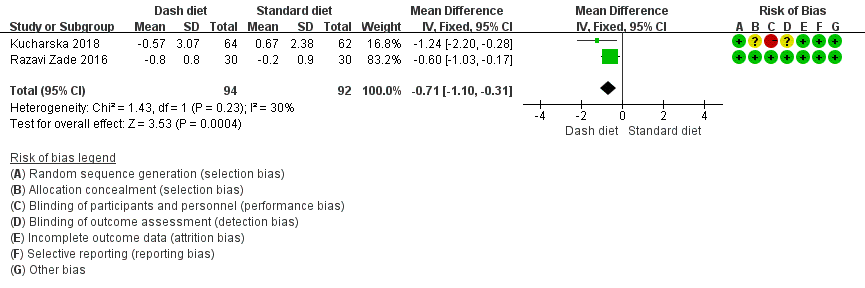


**Supplementary Figure 21.** Forest plot and bias risk for HOMA-IR.
